# A strategic approach to evaluating battery innovation investments

**DOI:** 10.1016/j.isci.2024.111075

**Published:** 2024-10-17

**Authors:** Rahul Malik, Koen Bertens, René-Pierre Allard, Katherine Thompson, Jennifer Hiscock, Cynthia Handler, Amanda Wilson

**Affiliations:** 1 Office of Energy Research & Development, Natural Resources Canada, Ottawa, ON K1A 0E4, Canada

**Keywords:** Energy policy, Engineering, Economics

## Abstract

Driven predominantly by public and private innovation, rechargeable batteries have, over a few decades, graduated from powering luxury consumer electronics to becoming one of the linchpins of the energy transition. Rapid adoption trends of batteries must accelerate to meet global net-zero targets for mobility and stationary storage, and will require making sound investments in battery innovation that deliver the most value. Because battery innovation is increasingly complex, multi-disciplinary, and subject to the coordination of stakeholders across academia, industry, government, and the broader public, building common and intuitive frameworks for understanding becomes critical to making progress. In this *Perspective*, we present and employ five conceptual, descriptive, technical, and social frameworks that, taken together, provide a holistic assessment of innovation opportunities in the battery sector. Finally, we illustrate their implementation as the foundation of the *Strategic Approach to Battery Innovation* pursued by the Government of Canada’s Office of Energy Research and Development.

## Graphical abstract

**Figure:**
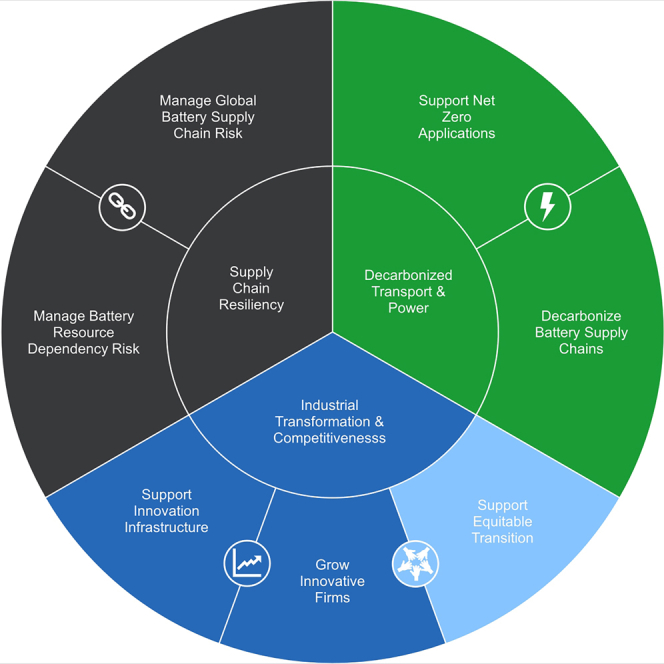

## Introduction

Several clean technologies will play important roles in the energy transition, but batteries are one of the few to have already made a significant difference by directly offsetting internal combustion engine vehicle and fossil fuel-powered electricity generation emissions. By accepting, storing, and releasing electrical energy on demand with minimal losses, batteries power the portable devices we use to work and communicate, and they are becoming the basis of a net-zero emissions economy enabled by the electrification of transportation and the storage of electricity from non-emitting sources. In 2023, electric vehicles (EVs) reached almost 18% of new car sales globally,^1^ and energy storage deployments approached 100 GWh with 21% compounded annual growth expected to the end of the decade.^2^

Even so, to achieve net-zero emissions by 2050, the pace of battery adoption needs to accelerate even faster. The rise of battery adoption in the past decade correlated with consistent price declines amounting to a 90% total cost reduction, a trend that was temporarily disrupted in 2022 when prices increased by 7% from rising battery input costs.^3^ While battery sales still increased in 2022 and after, illustrating resilient demand, prices must continue down a rapidly declining trajectory for batteries to support affordable decarbonization pathways across the full spectrum of transportation and net-zero community energy needs.

Innovation in technological performance was the biggest driver of battery price decline observed between the late 1990s and mid-2010s, with the largest share of this originating from research and development (R&D), followed by economies of scale of production.^4^ For road transport alone, staying on track for net-zero emissions by 2050 sees global cumulative battery demand increase 90-fold from 2023 to 2050,^1^ so the ability for the supply chain to meet demand for battery applications will be strained without new innovative solutions in place. Thus, while battery innovation got us this far, we have further to go with less time.

Battery innovation is complex and multi-faceted. Even more so today than in previous decades, it crosses disciplines and includes industries adjacent to batteries and other newcomers to battery innovation who increasingly have a greater voice in the conversation. These include multiple diverse stakeholders with varying degrees of battery experience and understanding, spanning academia, industry, government, and the broader public. There is a need to facilitate conversations inclusive of non-expert battery stakeholders to build the common understanding needed to think strategically and most effectively allocate investment resources to areas of battery innovation that will return the most value. To this end, we propose five conceptual, descriptive, technical, and social frameworks that, when taken together, provide a holistic assessment of battery innovation opportunities: (1) anatomy of a battery, (2) battery performance metrics and application requirements, (3) the battery value chain, (4) scaling batteries and technology readiness levels, and (5) battery sustainability.

In the following *Perspective*, we introduce and develop each battery framework, describe how to apply them, and conclude by illustrating their specific implementation by the Government of Canada’s Office of Energy Research and Development (OERD) within Natural Resources Canada (NRCan) in its *Strategic Approach to Battery Innovation* implemented for 2024–2035.^5^

## Framework 1: anatomy of a battery

**Figure 1:**
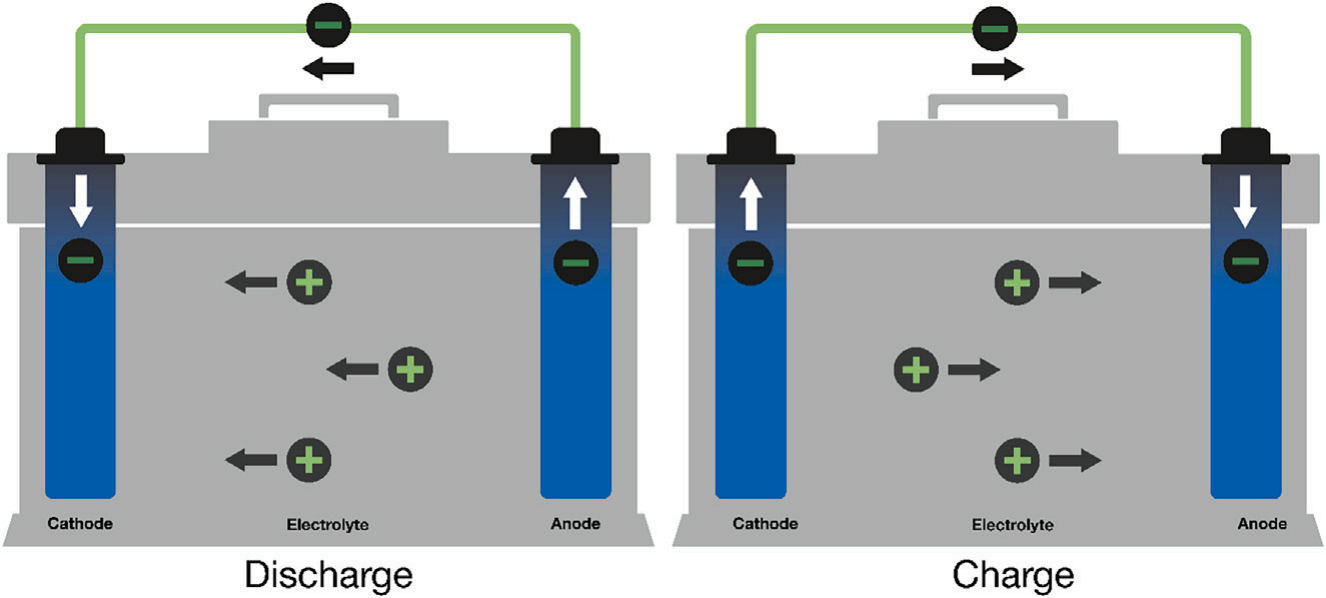
Schematic diagram of a battery

A battery is capable of accepting, storing, and releasing electricity through the selection, arrangement, and interaction of three main cell components—the *anode*, *cathode*, and *electrolyte* (described schematically in Figure 1, depicted in a closed cell architecture). In a lithium-ion (Li-ion) battery, for example, the energy is stored in solid electrode materials (the anode and cathode active materials) as Li is shuttled back and forth through the liquid electrolyte, which conducts Li ions (positive circles) but not electrons (negative circles). In charging and discharging, the electrons are shuttled back and forth between anode and cathode through the external circuit, providing electricity on demand. While the materials and arrangements of these main components differ between open and closed battery architectures,^6^^,^^7^ this framework is inclusive to all battery types.

While the aforementioned description of a battery may initially appear overly simplistic, it sufficiently captures key weaknesses and strengths of the technology. Figure 1 indirectly conveys the high materials intensity underlying batteries due to the strict relationship between the quantity of electrical energy that can be stored (as chemical potential) and the amount of active electrode material needed. In a Li-ion battery, for every electron stored at least one Li is needed, as well as at least one redox center in each active material, a role commonly served in the cathode by expensive critical battery metals like Ni and Co. While a full life cycle analysis across system boundaries is needed for a more precise comparison, a battery’s ability to reversibly charge and discharge, in some instances for thousands of cycles with high round-trip efficiency,^8^ provides an enormous advantage over fossil fuel combustion, which allows for just one-time energy conversion. Consequently, applications substituting fossil fuel combustion with batteries not only support the energy transition, but also substantially reduce the overall mining requirement when compared to the current fossil system.^9^

Consideration of the anatomy of a battery also helps frame the value proposition of multiple next-generation battery technologies. For example, the materials intensity inherent to batteries drives innovation in chemistries with substitutes for critical metals, such as sodium-ion^10^ and potassium-ion^11^ batteries, or iron- and manganese-rich cathode chemistries. Multivalent battery chemistries^12^ are compelling because they have potential to break the aforementioned one-to-one relationship between stored electron and shuttled ion (i.e., by achieving 2 electrons stored per Ca, Mg, or Zn ion shuttled compared to 1 electron stored per Li ion). Similarly, battery chemistries capable of multiple redox, for instance by storing 2 electrons per cathode transition metal (such as with Mn or V)^13^^,^^14^ or additionally storing electrons using anionic redox,^15^ break the one-to-one relationship between the number of required critical battery metal atoms and stored electrons. While the aforementioned approaches offer pathways to improve upon key aspects of current battery technology, they also introduce tradeoffs in battery performance metrics that must be negotiated to meet application requirements.

## Framework 2: battery performance metrics and application requirements

A battery’s end-use application can be framed by its requirements for *energy*, *power*, *safety*, lifetime, and *cost*.^16^ For example, a battery electric vehicle (BEV) has the general requirements shown in Table 1.

**Table 1:** Generalized requirements for BEV applications

Battery performance metric	Application requirement
Energy	Vehicle range and size determine the energy requirement.
Power	The charging time determines the input power requirement, and vehicle propulsion and size determine the output power requirement.
Safety	The cells and battery packs must surpass a minimum standard of safety to be roadworthy, crashworthy, and handled safely throughout the products’ lifetime.
Lifetime	The vehicle warranty, in years and kilometers driven, determines the lifetime requirement.
Cost	Reaching price parity, and ultimately undercutting incumbent internal combustion engine vehicles, determines the target battery cost, which is the largest contributor to overall BEV cost.

Other classes of vehicles also use Li-ion batteries, such as mild-hybrid (MHEV) or plug-in (PHEV) electric vehicles, but their requirements for each category shown in Table 1 are adjusted. For example, an MHEV battery does not need to provide vehicle propulsion, and a PHEV battery has a lower driving range requirement, so accordingly smaller batteries are more appropriately sized for these applications compared to a BEV. The MHEV, PHEV, and BEV must meet the same regenerative braking requirement (assuming the same size of vehicle and driving behavior), which requires the MHEV and PHEV batteries to receive the same input power as the BEV battery, but with smaller battery capacity. In other words, they need to operate at higher C-rates (i.e., the input or output power normalized by the energy capacity).

Requirements for stationary storage applications, including for those far-removed from Li-ion battery technology such as long-duration energy storage (LDES), also fall under the aforementioned categories. Unlike batteries for mobility applications, LDES costs compete with the much lower price of electricity rather than the price of an internal combustion vehicle, and cycling over long durations means much less power is needed relative to energy stored (consequently, very low C-rates). Operating at much lower rates compared to mobile battery applications also means accumulating fewer cycles in time, which translates to a lower cycle life requirement over the product lifetime.

Depending on the application, composite figures of merit involving combinations of the battery performance metrics in Table1 may be preferable to assess suitability for an application or compare performance. For example, for stationary storage, the levelized cost of storage (LCOS) and annuitized capacity cost (ACC) capture the cost over lifetime energy throughput (measured in $/MWh) and lifetime power (measured in $/kW-year), respectively.^17^

Battery materials, cells, and packs ultimately determine the end-use application’s performance envelope along the aforementioned categories, which must at minimum match or exceed the application requirements. Closing the gaps between performance and application requirements, while navigating the complex relationships and tradeoffs between each of the key battery performance metrics, defines the battery innovation challenge.

For evaluating innovative battery concepts, consideration of application requirements at the outset provides accountability, guidance for validation, and a bridge between early-stage R&D and final implementation in a product for sale. In early-stage research, validation against application requirements motivates following standardized reporting guidelines.^18^^,^^19^^,^^20^ With further technology maturity, requirements are reported by commercial bodies like the United States Advanced Battery Consortium (USABC)^21^ and the European Council for Automotive R&D (EUCAR)^22^ which set targets that are relevant to the industry.

## Framework 3: the battery value chain

**Figure 2:**
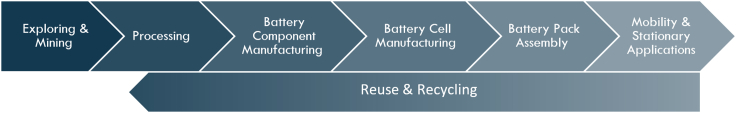
Battery value chain framework following stages of production and operation

In parallel to the rise of Li-ion batteries in mobility and stationary applications over this decade, a global battery supply or value chain has developed according to stages of production and operation. While there are various depictions and levels of detail that can be included in the battery value chain, we refer to a high-level representation with seven major segments depicted in Figure 2.

Key battery inputs for active materials are found in low concentration in ores (in single-digit percentages), which are mined and processed with considerable time, energy, and cost to more than 99% purity to become suitable as battery-grade reagents. While batteries for differing applications may require different mineral inputs from across the globe, all material inputs are required to be extracted and processed, or in the case of reuse and recycling, only re-processed. Next, battery components including anode, cathode, electrolyte, and other inactive components (such as aluminum and copper foils, additives, etc.) are intricately synthesized and mass produced, then integrated together precisely in cell manufacturing. The resulting cells can be considered semi-finished products, and at this stage, performance can be readily evaluated against application requirements. Multiple cells are physically arranged and wired in series and parallel in the battery pack, which employs an electronic battery management system (BMS) to govern the safe operating limits of the cells. Packs are integrated into end-use applications (here we focus on mobility and stationary storage) and cycled to end-of-life (EOL) in operation. Batteries may be re-used in a second life application with more relaxed performance requirements compared to their first use. Finally, battery recycling (and broader circularity efforts) is necessary to extract maximum value and minimize waste, which closes the loop on the value chain.^23^

The battery value chain is a descriptive framework that follows the entire product life cycle through time. It captures the direction of materials and energy flows toward the production and operation of batteries, and these correlate with economic value creation and environmental footprint. Also, key stakeholders and their relationships in the global battery ecosystem can be categorized by value chain segment with relative ease, but this will become more challenging with the growing trend toward vertical integration bridging multiple segments.^24^ Analyzing and mapping the battery value chain reveals gaps and opportunities where battery innovation cognizant of upstream and downstream linkages can provide most value.

## Framework 4: scaling batteries and technology readiness levels

The standardizable, modular, and mass-producible character of battery technology has helped facilitate today’s increasing deployment and rapidly falling costs,^25^ and future innovations would be best served by retaining and building upon these qualities to continue and accelerate the trend. Batteries now have implications across a range of scales spanning more than eight orders of magnitude, which motivates the need to have a firm footing in battery numeracy to guide thinking around battery innovation to meet the deployment challenge ahead.

At the application level, for example, a large BEV battery pack can contain 100 kWh of energy capacity. A large stationary storage installation today can be 1 GWh (equivalent to 10,000 large BEV battery packs), and a modern battery gigafactory can produce around 50 GWh a year (enough for 500,000 large BEV battery packs per year).

The global demand for batteries in 2023 was just over 1 TWh (across all sectors but dominated by passenger EVs), approximately the annual output of 20 modern battery gigafactories. In the net-zero scenario, annual global Li-ion battery demand will need to grow to almost 10 TWh in 2035, representing an almost 10-fold increase in 12 years.^1^

**Figure 3:**
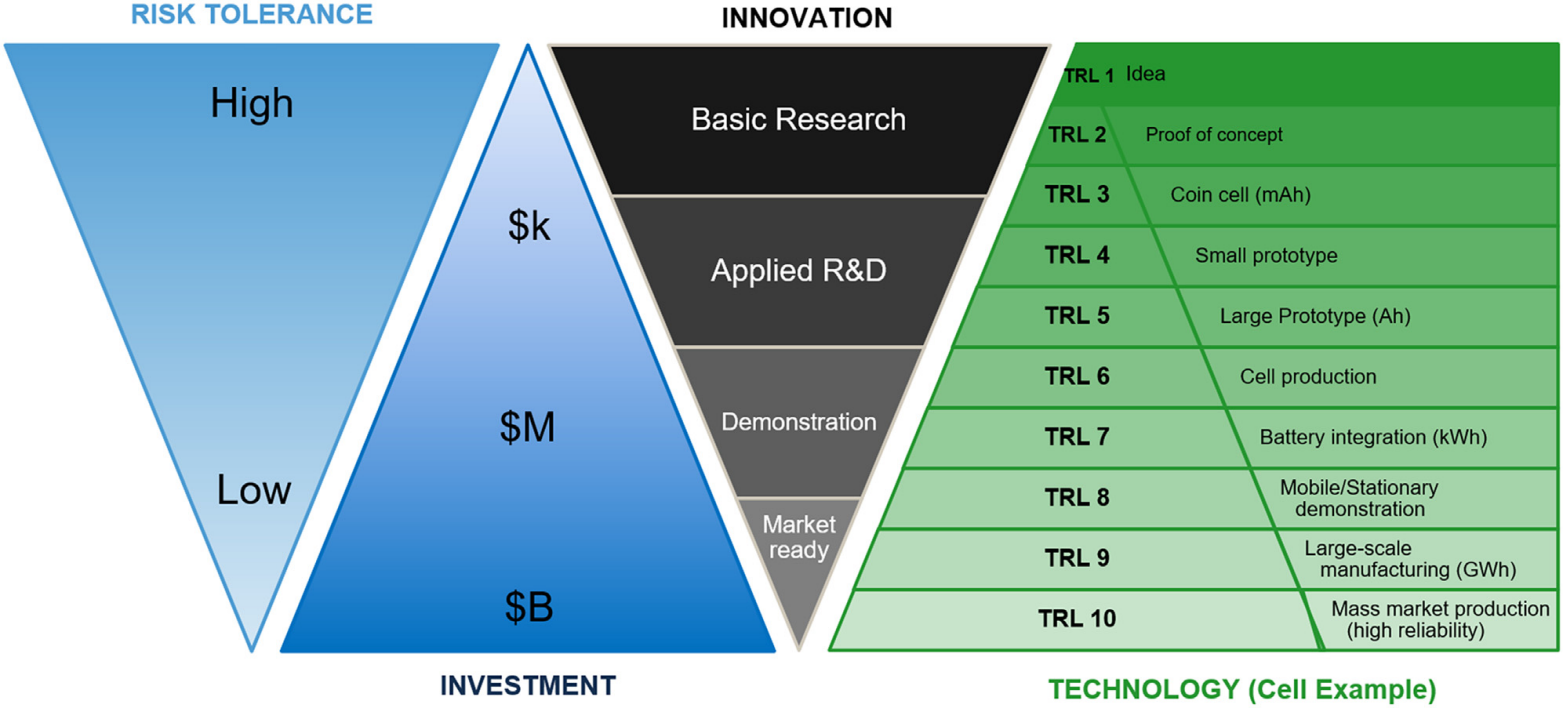
Technology readiness level scale for BEV applications, adapted from Frith et al.

Technology readiness level (TRL) is widely adopted by government innovation programs as a semi-quantitative and generic measure of a technology’s maturity and scale. While other frameworks such as innovation roadmaps can also be used to chart progression in innovation,^26^^,^^27^ TRL is helpful to evaluate progression from basic research (TRL 1–2) through applied R&D (TRL 3–4), prototyping and validation (TRL 5–6), field testing and demonstration (TRL 7–8), and adoption (TRL 9+). TRL scales for innovations along specific battery chain segments, for instance for cell^28^ (shown in Figure 3) and component development,^29^ correlate with increasing scale of deployment, battery size, and required level of investment. Conversely, innovation at lower TRL has a higher tolerance to risk of failure, while innovation at high TRL is more sensitive due to the high level of investment needed for a large-scale experiment.

## Framework 5: battery sustainability

**Figure 4:**
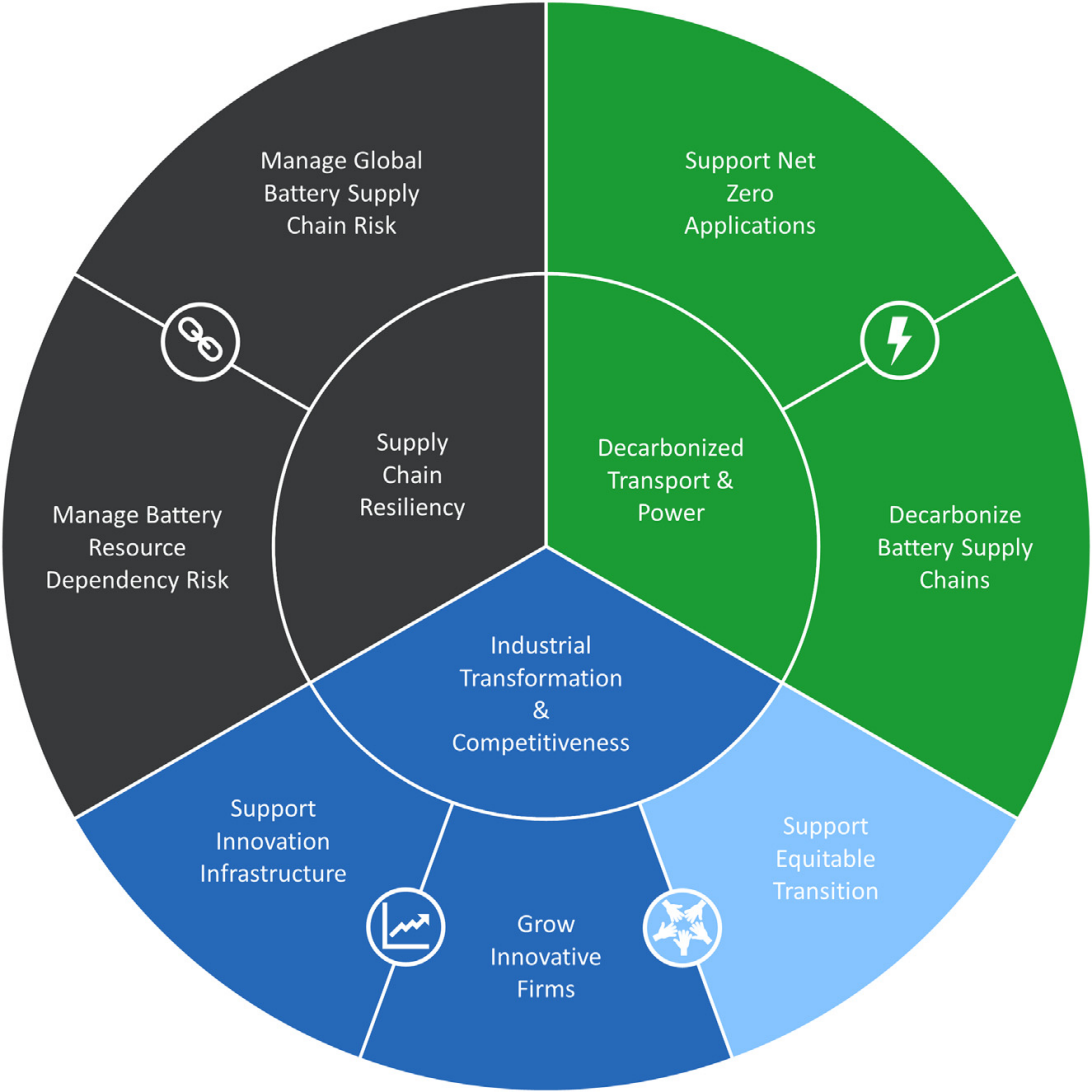
Battery sustainability framework

The final framework in Figure 4 was derived from the well-known energy trilemma framework.^30^ It recognizes that battery innovation is not solely a tool to address technical challenges. Rather, batteries serve as a key technology of the global energy transition, and therefore, strategic thinking along *economic*, *environmental*, and *security* principles must factor prominently into battery innovation programming decision-making.

The crucial value of batteries in the net-zero economy is to provide an affordable energy storage alternative to fossil fuels and extend the reach of electrification in the economy, ultimately powered by non-emitting energy sources. The value of battery innovations will ultimately be measured by their impact on the environment, which motivates the importance of accurate and useable life cycle analysis tools that capture a fuller picture of environmental footprint of batteries across the value chain.

The global battery ecosystem is emerging as a major economic driver, underpinning not only the establishment and growth of massive battery materials, components, and cell production industries, but also the future of the automotive and extractive industries. Within the portfolio of all clean energy technologies needed to get to net-zero emissions, batteries are a high maturity and safe investment with a large established market demand and high projected growth, and consequently several jurisdictions are making investments to anchor a part of the future battery innovation and industrial base. The need to establish industrial transformation and competitiveness is balanced by the need for an equitable transition that generates high-quality, fairly compensated jobs and maintains energy affordability.

The outsized global economic and environmental implications outlined previously elevate batteries and battery innovation to issues of national security. Battery demand is large, growing, and global, while the battery value chain is currently dominated by a few companies and state actors. Today’s battery input materials are also concentrated geographically, and multiple countries have developed and executed battery-specific industrial strategies to reap the benefits from having control of the battery supply chain.

A country’s ability to be self-sufficient and maintain autonomy now depends on its ability to serve domestic battery needs, especially for critical infrastructure such as grid and defense applications. Against this backdrop, homegrown innovation in batteries, including strategies for bringing them to market, protecting intellectual property, and collaborating internationally, becomes a critical lever for supply chain resilience and economic competitiveness.

## Applying the battery frameworks: a strategic approach to battery innovation in Canada

Collectively, the aforementioned frameworks presented can be applied to support accurate and holistic assessments of batteries and battery innovations. They were selected not only for their simplicity, as accessible entry points for non-experts seeking to make quick but informed decisions related to batteries, but also for their application to more complex concepts and nuances of batteries in a broader context. In practice, investment opportunities in battery innovation can be evaluated against each framework to stay consistent in investment approaches and identify opportunities that create value. The frameworks also provide a means to compare the relative strengths, weaknesses, and common objectives of widely varying battery innovations (e.g., across the battery value chain, technology readiness levels, chemistries, cell designs, etc.).

The OERD leads the Government of Canada’s efforts in delivering energy research, development, and demonstration (RD&D) funding, and has provided over $1.4 billion for projects since 2016. OERD targets the most impactful areas of energy innovation, including batteries, to maximize environmental benefits and economic outcomes. Leveraging fifty years of experience and unique science and technology expertise, OERD invests in research facilities across the Government of Canada, as well as a wide range of Canadian small and medium enterprises, industry, utilities, academia, and other organizations, all in support of Canada’s energy innovation and climate goals. OERD employs a systems-based approach to identify areas of focus based on technological, market, regulatory, and policy gaps and barriers. Using the battery frameworks as a foundation, OERD has developed the *Strategic Approach to Battery Innovation* to guide funding and non-funding support for the Canadian battery sector.

As a mid-sized open economy with a top ten nominal gross domestic product and nascent battery ecosystem, Canada must leverage its unique battery strengths to compete on the world stage. In addition to a generous resource endowment of nearly all battery inputs (including Li, Ni, Co, and graphite resources), more than 80% of electric power in Canada comes from non-emitting sources. Backed by a long track record of major contributions that have advanced battery technology, Canada also has established a strong battery innovation pedigree.

Taken together, OERD sees a window for Canada to lead in producing and operating homegrown, sustainable, and innovative batteries and technologies. Accordingly, Table 2 highlights specific battery innovation opportunities targeted by OERD’s funding and non-funding supports along the three principles of battery sustainability. Battery frameworks help identify opportunities for the largest innovation impact and reveal unaddressed gaps.

**Table 2:** OERD battery innovation opportunities

Battery innovation opportunity	Description	Frameworks represented
Materials substitution, abundant battery chemistries, and affordable batteries	Battery innovation via substitution of costly materials and components with cheaper, more abundant, and geographically diverse alternatives while maintaining or improving performance.	• Battery sustainability • Anatomy of a battery
Application-guided battery innovation	Consideration of specific end-use application requirements and Canadian operating conditions at all stages of the battery innovation process to accelerate path to market.	• Battery performance metrics and application requirements
Vertical integration, processing, and advanced manufacturing of batteries	Battery innovation using upstream and downstream linkages to yield efficiencies spanning segments of the value chain, both in terms of reductions in the material and energy intensity of production and increase in production throughput and quality.	• Battery value chain • Anatomy of a battery
Battery data and modeling approaches	Computation and data approaches to battery innovation to accelerate the pace of insight generation and translation from concept to market implementation.	• Scaling batteries and technology readiness levels • Anatomy of a battery
Battery circularity	Innovation to minimize the generation of waste across the battery value chain including the generation of emissions in the manufacturing and upstream processes of battery production.	• Battery value chain • Battery sustainability
Battery standards and regulations	Innovation to accelerate the generation, development, and adoption of standards and regulations of batteries to advance interoperability, safety, traceability, and environmental practices	• Battery sustainability • Battery performance metrics and application requirements
Battery services and business models	Innovation in support of new market development and new use cases of batteries including for climate adaptation, long duration energy storage, batteries as a service, battery swapping, and vehicle-to-X (V2X).	• Battery value chain • Battery performance metrics and application requirements

Innovation that enables abundant and highly accessible battery inputs is primarily intended to improve affordability by prolonging the downward price trajectory of batteries. Where possible, meeting battery demand with cheaper inputs complements Canada’s strength in critical minerals resources and development, freeing up those resources for deployment in the applications where they are needed most. Combined with recent major investments across the battery value chain, including in cathode manufacturing, cell manufacturing, and BEV assembly, Canada is pursuing a “mines to mobility” strategy,^31^ which also presents a unique opportunity for innovation in vertical integration and advanced manufacturing. With local supply of battery inputs and availability of cheap non-emitting electricity for manufacturing and charging, innovation in battery circularity in Canada opens the possibility for minimizing the environmental footprint of batteries. Finally, operating batteries in the Canadian environment, namely in large but sparsely populated regions that encounter extreme low temperatures, presents unique challenges that require innovation to overcome.

**Figure 5:**
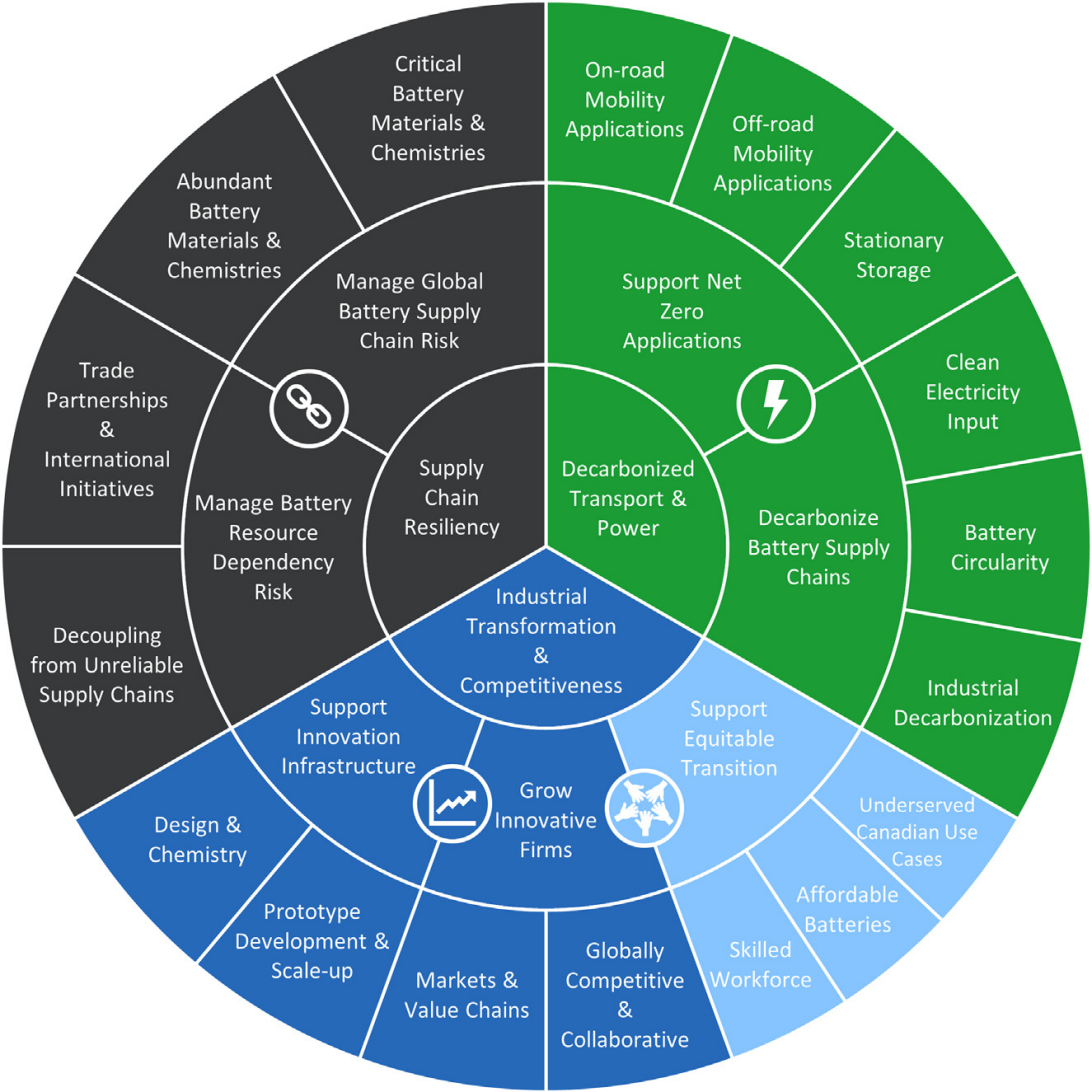
Application of the battery sustainability framework to OERD’s *Strategic Approach to Battery Innovation*

Highlighted in Figure 5, which is derived from the battery sustainability framework (shown in Figure 4), the *Strategic Approach to Battery Innovation* addresses specific elements of the Canadian battery ecosystem along the three principles of sustainability and follows three pillars: (1) support innovation that accelerates battery value chain decarbonization, security, and competitiveness; (2) support the development of world-class battery innovation infrastructure in Canada; and (3) strengthen the network of Canadian battery innovators and help grow world-class Canadian firms in the battery value chain.

OERD operationalized the *Strategic Approach to Battery Innovation* in March 2024 and launched the Battery Industry Acceleration (BIA) call for proposals under NRCan’s Energy Innovation Program (EIP).^32^ Drawing from the battery sustainability framework, the BIA call is designed primarily to advance pillars (1) and (3). Furthermore, the call’s eligibility, scoping, and priority areas are based on the battery frameworks and innovation opportunities, with support targeted toward for-profit firms pursuing RD&D projects focusing on materials, components, cells, and packs in the battery value chain.^33^

Advancing pillars (1) and (2) of the *Strategic Approach to Battery Innovation*, OERD also invested in Dalhousie University in February 2024 to help support the establishment of the Canadian Battery Innovation Center (CBIC).^34^ Envisioned as innovation infrastructure available to both industry and academic battery researchers seeking to construct next-generation battery cells at the highest industry standards, the CBIC’s objectives are not only to advance key battery technologies toward commercialization, but also to provide industry-oriented training in battery cell manufacturing to support the development of a sustainable domestic battery innovation ecosystem.

## Conclusion

Determining appropriate measures for supporting innovation is a dynamic problem, especially for government, particularly in rapidly evolving industries like the battery sector. Not only can innovation advance technology maturity, but it can also break through market, regulatory, and other institutional barriers to the energy transition. The five frameworks presented in this *Perspective* help evaluate attributes of different innovation opportunities in both Canada’s emerging battery sector and the international ecosystem. Mapping segments of the battery value chain to policy drivers for the energy transition, this framework approach highlights the interdependency of problems and potential solutions in the sector. OERD’s *Strategic Approach to Battery Innovation* is informed by the frameworks and serves as a guideline to more transparently develop and apply evaluation criteria for innovation opportunities and results analysis applicable across battery chemistries or applications.

## Acknowledgments

This project was financially supported by the 10.13039/501100007178Office of Energy Research and Development in 10.13039/501100000159Natural Resources Canada. We would like to acknowledge this support, as well as valuable feedback from René Silva Jimenez, Susan Hwang, and Alan Sovran from the 10.13039/501100007178Office of Energy Research and Development in 10.13039/501100000159Natural Resources Canada.

## Author contributions

Conceptualization and investigation, R.M.; writing – original draft, R.M., K.B., and J.H.; writing – review, editing, and revision, R.M., K.B., R.-P.A., K.T., J.H., C.H., and A.W.; visualization, R.M., K.B., K.T., and J.H.; supervision, C.H. and A.W.

## Declaration of interests

The authors declare no competing interests.
